# Comparison of Quantitative Detection Methods Based on Molecular Fluorescence Spectroscopy and Chromatographic Techniques Used for the Determination of Bisphenol Compounds

**DOI:** 10.3390/ijms221910569

**Published:** 2021-09-29

**Authors:** Joanna Orzel, Pawel Swit

**Affiliations:** Institute of Chemistry, University of Silesia in Katowice, 9 Szkolna Street, 40-006 Katowice, Poland; pawel.swit@us.edu.pl

**Keywords:** BPA, BPS, BPF, molecular fluorescence spectroscopy, HPLC-DAD, HPLC-FLD

## Abstract

Analytical methods using the fluorescence properties of bisphenols (BPA, BPF and BPS) and their complexes with β-cyclodextrin and methyl-β-cyclodextrin were developed. The methods were applied for the analysis of thermal paper and canned food. Their performance was compared with a standard HPLC approach with a diode array and fluorescence detections. For comparison purposes, basic validation parameters (linear range, limit of detection, sensitivity, precision) were evaluated. It was concluded the developed methods facilitate fast and cost-effective determination of three bisphenol species in liquid samples, similar to the HPLC performance. They are also environmentally friendly. BPA, BPF and BPS can be routinely determined with the presented approach.

## 1. Introduction

Polymers surround us constantly and are ubiquitous. Personal hygiene products, electronics, food containers, medical supplies, and many more make our life easier and more comfortable. It is convenient and cheap to use these kinds of commodities, nevertheless, it can be hazardous to use them regularly. Although it takes a long time to fully biodegrade a polymer, the products of this process are constantly polluting the environment [1,2]. The impact of these pollutants on every living organism is an issue we are facing and desperately need to challenge.

Bisphenols (BPs) and their derivatives are a group of the most prevalent polymer-origin pollutants of the environment and of the products we eat, drink, and use daily [3,4,5]. Bisphenols are a family of compounds containing two phenolic rings connected by a carbon atom (or a sulphur atom). They are modified by aromatic rings or connecting atom substituents [6]. Bisphenols are used as epoxy resins and polycarbonate precursors [7]. These polymers find varied applications: coatings of water pipes and cans designated for food and drinks (e.g., vegetables, fruits, soft drinks, and beer), food storage containers, CDs and DVDs, and many more [8]. Thermal paper production (e.g., that used for sales recipes or tickets), dental fillings, and epoxy glues are yet other applications of BPs [9]. Considering the extensive use of bisphenols, they can be easily transferred into our bodies. Owing to the similarity in the structure of BPs and some hormones, they act as endocrine system disruptors. Bisphenols are recognized as xenoestrogens, causing cardiac, neural and reproductive dysfunctions, obesity triggers, and fetal health disruptors [10,11,12].

Bisphenol A (BPA, 4,4′-(propane-2,2-diyl)diphenol) is the most popular of the BPs. Its impact on the wellbeing of people and animals has been extensively studied [10,13,14]. Its use in the manufacturing of baby products is banned in many countries [15]. Thermal paper production with BPA is also restricted in the European Union, Japan and many other places all over the world [9,16,17]. Unfortunately, BPA is substituted with other BPs. The most popular substituents are bisphenol S (BPS, 4,4′-sulfonyldiphenol) and bisphenol F (BPF, 4,4′-methylenediphenol). Their impact on our health and environment is reported to be similar to BPA, but they are not yet legally restricted [18,19].

Due to their adverse health issues, we should monitor BPs concentration in food, water and the body fluids of exposed workers (e.g., cashiers). This requires quick and inexpensive procedures. Several approaches have been developed for the qualitative and quantitative analyses of BPs [17,20]. The most commonly used is high performance liquid chromatography (HPLC) with various detectors, i.e., UV detectors, diode array detectors (DAD), fluorescence detectors (FLD), or with mass spectrometry (MS). Less popular is HPLC with electrochemical detection [21]. BPs’ separation (after derivatization) is also performed with gas chromatography [22]. Although these methods offer low detection limits (down to ng∙mL^−1^), they are expensive, time-consuming and require trained staff. In the literature, few methods based on direct sample measurements (without chromatographic separation of analytes) have been described. Due to low concentrations of BPs in samples, various adsorbents have been developed to preconcentrate the BPs with solid phase extraction (SPE) [23]. Electrochemical detection of bisphenols using modified electrodes (including aptamers or molecularly imprinted polymers) is another popular method [24,25]. These kinds of approaches offer even lower detection limits than the HPLC methods, but they are difficult to transfer for commercial purposes. The ELISA methodology has also been applied for the analyses of bisphenols [17]. Another alternative method for the direct evaluation of BPs is molecular fluorescence spectroscopy (MFS). BPA, BPF and BPS are described in the literature as fluorescent molecules [26,27]. Vidal et.al., studied the fluorescence properties of BPA and its complexes with cyclodextrins (CDs) [28]. It has been reported that methyl-β-cyclodextrin (m-β-CD) enhances the fluorescence properties of BPA in aqueous media, and thus, the limit of detection (LOD) decreases over 30 times. BPA complexes with β-cyclodextrin (β-DC) were also studied, but detailed data have not been presented. To the best of our knowledge, the impact of cyclodextrin compounds on fluorescence properties of BPS and BPF have not yet been presented in the literature. There is also a lack of performance comparison between the MFS and HPLC methods.

The simplicity of sample preparation, measurements and data handling are the main advantages of MSFs. In this study, the quantitative detection of the three most popular BPs (BPA, BPF and BPS) in the appearance of CDs was explored. The influence of β-CD and m-β-CD on the analytical parameters (linear range, limit of detection, sensitivity, precision) are examined and compared with results delivered for BPs detection without the presence of the CD compounds. For evaluation purposes, BPs were simultaneously quantitated using well established HPLC-DAD and HPLC-FLD methods. The developed methods are applied for the analyses of real samples, i.e., thermal paper and canned food samples.

## 2. Results and Discussion

### 2.1. Exploration of Fluorescence Properties

**Figure 1:**
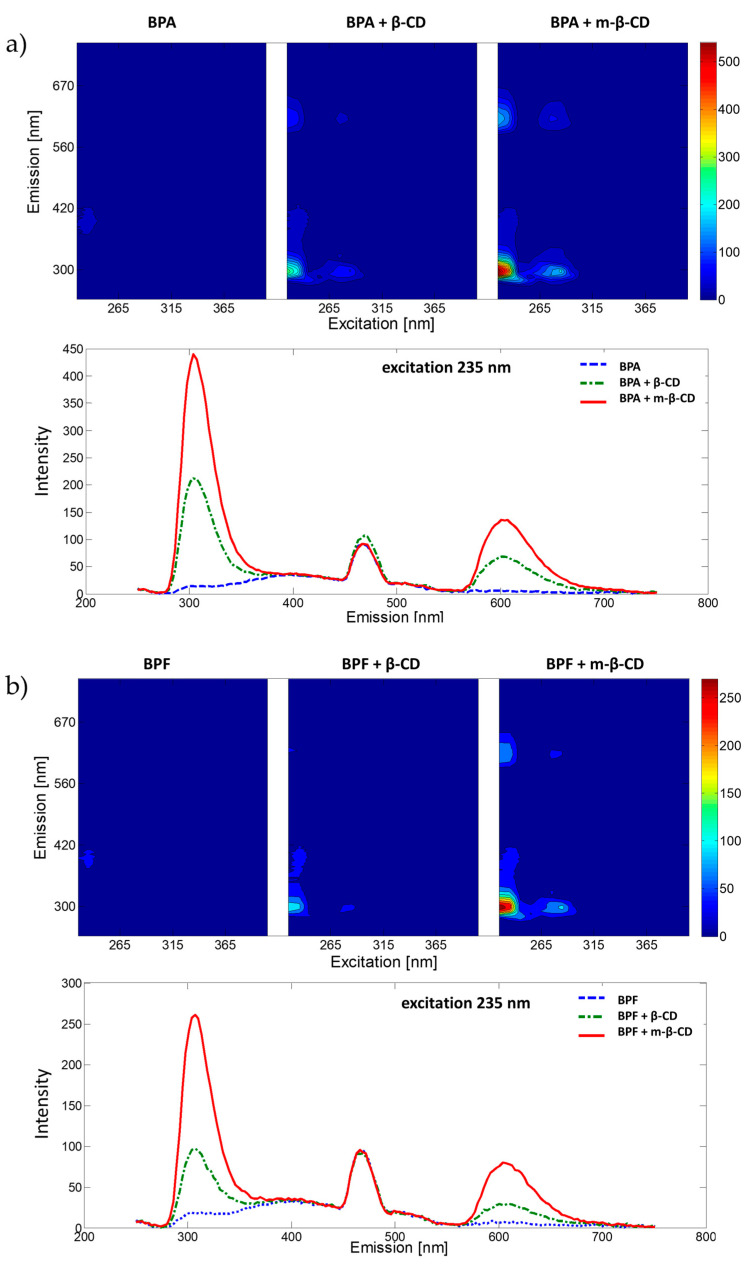

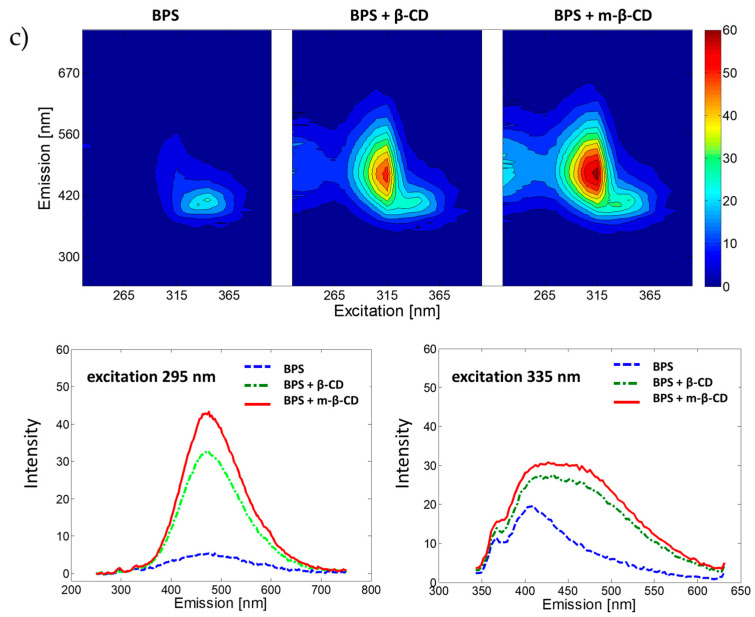
EEMs and fluorescence emission spectra for selected excitation wavelength of bisphenols with m-β-CD, β-CD or without CD molecules in phosphate buffer (pH = 7.00) for (**a**) BPA, (**b**) BPF and (**c**) BPS.

The fluorescence properties of BPA, BPF and BPS and their complexes with β-cyclodextrin and m-β-cyclodextrin were analysed in water solutions (under pH = 7.00, phosphate buffer was used). Samples were excited from 225 nm to 405 nm with 10 nm intervals, and for each excitation wavelength the emission spectrum was recorded (measurement range 230–700 nm, data interval 3 nm) to collect the EEMs presented in Figure 1. For all studied BPs, the addition of β-CD and m-β-CD increases the fluorescence signal (compared to the fluorescence signal registered for BP without the CDs). Previous studies [28] show that the cyclodextrin compound hosts the BP compound in an inclusion complex favouring fluorescence emission. Excitation wavelengths resulting in significant analytical information were selected for each analysed system using recorded EEMs, see Figure 1. Selection of excitation wavelengths was confirmed with absorption spectra registered using a UV-Vis spectrophotometer. Spectra are included in Supplementary Materials (Figure S1). The influence of pH on the fluorescence spectra of selected complexes was also studied. Obtained results (see Figure S2) confirmed the selection of pH = 7.00 for further analyses.

Compositions of the inclusion complexes were studied using Job’s method. Fluorescence properties of a series of equimolar solutions for BP’s and cyclodextrins were recorded under selected earlier excitation and emission conditions. For each studied complex, the molar ratio of BP:cyclodextrin compound was found to be 1:1. Obtained Job’s plots are included in Supplementary Materials (see Figure S3).

### 2.2. Chromatographic Analysis

The optimal conditions for chromatographic separation were found using the CCD approach. We based this on previous research [29] to optimize an in-house method for BPA, BPF and BPS separation, thus a flow rate ranging from 0.5 mL∙min^−1^ to 1.35 mL∙min^−1^ and a mobile phase composition of water:ACN were considered with a fixed column temperature at 30 °C. A total of 20 experiments were carried out (see Table 4 for details). A second-order model was fitted to the obtained data. The analysis of variance (ANOVA) was used for the evaluation of the regression coefficients significance (see Table 1).

**Figure 2:**
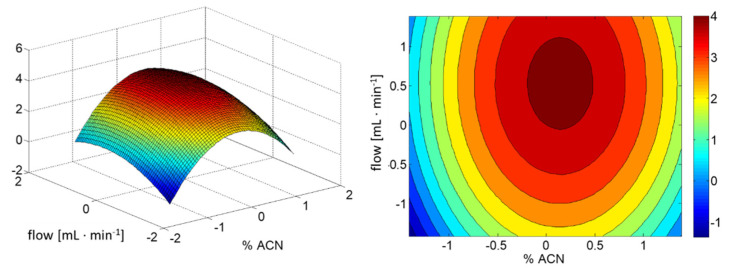
The response surface obtained from the CCD. A description of the coded parameters is presented in Table 4.

On the basis of the results presented in Table 1, it was concluded that the interaction of studied factors is insignificant for optimal separation of BPs. Using only significant factors, the response surface depicted in Figure 2 was constructed.

**Figure 3:**
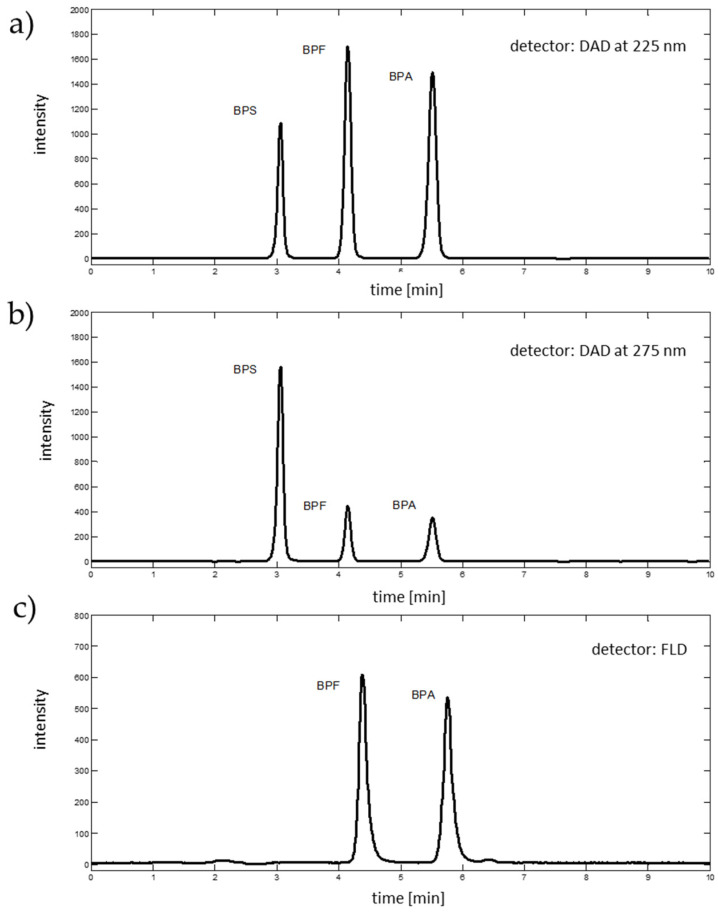
Chromatographic signals (**a**) extracted from DAD data at 225 nm, (**b**) extracted from DAD data at 275 nm, and (**c**) registered with fluorescence detector, excitation 230 nm and emission 300 nm.

The best separation of analytes was obtained with a flow rate of 1.35 mL∙min^−1^ and a mobile phase of acetonitrile:water ratio of 50:50 (*v*/*v*). With these conditions, studied analytes were eluted at 3.06 min (BPS), 4.15 min (BPF) and 5.51 min (BPA). For calibration purposes, two absorption wavelengths, resulting in chromatographic signals with the highest peak areas, were selected from the DAD spectra (i.e., 225 nm for BPA and BPF, and 275 for BPS), see Figure 3. The fluorescence detector (excitation 230 nm, emission 300 nm, sensitivity medium) facilitates the detection of BPF and BPA, and was eluted at 4.38 and 5.76 min, respectively (see Figure 3). Fluorescence of BPS was too weak to detect with applied fluorescence detector parameters.

### 2.3. Analytical Methods

Calibration models based on the fluorescence signals were constructed for each of the studied BPs in three systems:

(1) selected BP in phosphate buffer,
(2) selected BP and β-CD (1:1 molar ratio) in phosphate buffer (pH = 7.00),
(3) selected BP and m-β-CD (1:1 molar ratio) in phosphate buffer (pH = 7.00).

The systems were characterized by linear calibration curves (constructed using seven analytical standards, each standard was prepared in triplicate and measured three times) and basic validation parameters. Data were recorded for distinguished excitation wavelengths selected with 5 or 10 nm slits for excitation and emission, detector sensitivity set as 600 V and a scan rate of 360 nm∙min^−1^. Spectra registered using the 10 nm slits were characterized with a higher signal to noise ratio than those registered with the 5 nm slits. Data obtained with wider slits also resulted in calibration models with better analytical parameters. All results presented in this paper were obtained with the application of 10 nm slits.

Similar fluorescence properties characterize BPA and BPF. Excited at 235 nm, both exhibit concentration sensitive emission in two spectral ranges (with maxima around 300 and 600 nm, see Figure 1). Thus, calibration curves were constructed with emission registered at 300 and 600 nm for BPA systems and 304 and 604 nm for BPF systems. Comparing obtained results, better sensitivity, LOD and linearity was exhibited by models based on emission data recorded at 300 or 304 nm for BPA and BPF, respectively. The basic parameters of these models are summarized in Table 2. Graphical presentation of obtained calibration curves is provided in Supplementary Materials, Figure S4a,b.

According to the BPS systems, two concentration sensitive emission ranges were also recognized. However, this time they are concentration relevant. For low concentrations (up to 15 µg∙mL^−1^), samples excited at 295 nm emit light with a maximum at 471 nm; for high concentrations (up to 300 µg∙mL^−1^), samples excited at 335 nm emit light at 402 nm. Using the data, calibration models for the BPS systems were constructed and characterized with parameters included in Table 2. Graphical presentation of obtained calibration curves is provided in Supplementary Materials, Figure S4c,d.

Simultaneously, calibration models were constructed using the chromatographic data. Linear ranges of calibration curves were evaluated with seven analytical standards (measured in triplicates). Areas of peaks representing BPA and BPF were computed using chromatograms extracted from HPLC-DAD signals at 225 nm, and HPLC-FLD signals recorded for excitation at 230 nm and emission at 300 nm. Calibration data for BPS were extracted from HPLC-DAD signals at 275 nm. Peak areas were computed using an in-house routine with Matlab 2012 software. The summary of obtained results is in Table 2.

An overall conclusion is the introduction of β-CD and m-β-CD results with increased detection sensitivity of the three studied BPs (expressed as the slope value). For BPA and BPF, m-β-CD provides the best sensitivity, whereas for BPS, β-CD and m-β-CD result in similar outcomes. The LOD values are, however, independent of the presence of the selected cyclodextrins in the mixture. Fluorescence spectroscopy facilitates the detection of BPS in a wide range of concentrations (up to 0.3 mg∙mL^−1^). For low BPS concentrations (below 15 μg∙mL^−1^) samples should be excited at 335 nm, and its emission registered at 400 nm. In contrast, excitation at 295 nm with emission at 471 nm is recommended for higher BPS concentrations. For all developed methods, the introduction of CDs molecules increases precision (expressed as relative standard deviation (RSD)) of BPs’ evaluation. Results show that the HPLC with UV (or DAD) or fluorescence detection results in ten times lower LODs and broader linear ranges of analytical methods (up to 1.12 mg∙mL^−1^) than those based on the MFS without chromatographic separation. Still, the MFS detection of BPs (when complexed with cyclodextrins) results in satisfactory analytical outcomes. Performance of the developed approaches was tested for real samples.

The HPLC analysis was performed firstly to evaluate presence and recognize BPs. Detailed information on the preparation of samples selected for analysis is provided in the Materials and methods section. The TP samples were diluted with ethanol (1:4 for the DAD and 1:10,000 for the fluorescence detector). The canned food samples were analysed without dilution. For both types of samples, a 10 μL injection was fixed in each chromatographic run. All samples were examined in triplicates following the developed analytical method. BPS and/or BPF were detected in the TP samples, see Table 3. It is in agreement with the limitations of BPA application in thermal paper production introduced in the UE [9]. Only one BP type (BPA) was detected in canned food samples, see Table 3. On the basis of the constructed calibration models, concentrations of BPs in samples were evaluated.

Concentrations of the detected BPs were evaluated with the developed fluorescence approach. The BP-m-β-CD complexes were used for analysis since they exhibit the most satisfactory analytical performance. A standard solution of m-β-CD in water was prepared (0.23 μg∙mL^−1^) for the procedure. A total of 10 μL of TP extract or 1 mL of canned food samples were mixed with 1 mL of m-β-CD and filled with phosphate buffer to the volume of 10 mL. All samples were prepared in triplicates. Fluorescence spectra were registered for samples in spectral ranges suitable for BPs previously detected with the HPLC method. Concentrations of BPs in tested samples, evaluated with the developed calibration curves, are summarized in Table 3. Interference of possible background molecules (e.g., amino acids or dyes), present in real samples, with the fluorescence signal emitted by the analysed BPs is possible. The standard addition method (SAM) can be applied to overcome that issue. We performed the SAM method for the studied samples. Obtained results were in agreement with the results from the applied external calibration (EC). Since the EC approach is less laborious and requires less reagents than SAM, only the EC results are shown.

The BPs’ concentrations ascertained by the HPLC and MFS approaches were on corresponding levels. BPF in TP sample no. 1 was not detected with the HPLC-DAD approach. It can be concluded the method using MFS direct measurements is suitable for routine analyses without loss of sensitivity.

## 3. Materials and Methods

### 3.1. Reagents

Chemicals used for the analyses were of the highest purity. The following reagents were used: H_2_O (LiChrosolv, LC gradient, Darmstadt, Germany), acetonitrile (LiChrosolv, LC gradient, Darmstadt, Germany), ethanol (LiChrosolv, LC gradient, Darmstadt, Germany), BPA (Sigma-Aldrich, 98% purity, St Louis, MO, USA), BPS (Sigma-Aldrich, 98% purity, Beijing, China), BPF (Sigma-Aldrich, 98% purity, Tokyo, Japan), methyl-β-cyclodextrin (Sigma-Aldrich, 98% purity, Bratislava, Slovakia), β-cyclodextrin (Sigma-Aldrich, 98% purity, St. Louis, MO, USA). K_2_HPO_4_, KH_2_PO_4_ (Chempur, p.p.a., Piekary Śląskie, Poland).

Stock solutions of BPA, BPF and BPF (2 mg∙mL^−1^) in ethanol, and β-CD (0.045 mg∙mL^−1^) and m-β-CD (0.052 mg∙mL^−1^) in water were prepared and stored in a fridge for further analyses.

Three thermal paper samples and three canned food samples were selected for the experiment. Of the thermal paper (TP) samples, no. 1 was a clear thermal paper from a local distributor, while no. 2 and no. 3 were receipts collected from local supermarkets. Canned food samples (corn, peas and jack fruit) were purchased in a local store.

### 3.2. Fluorescence Spectroscopy

A Varian Carry Eclipse fluorimeter (Varian Inc., Mulgrave, Australia) was used in the experiment. Spectra were registered as excitation–emission fluorescence matrices (EEMs) for exploration purposes, with an excitation of 225–400 nm, Δ = 10 nm, emission 250–750 nm, Δ = 3 nm and as emission spectra recorded at a single excitation wavelength for quantitative evaluation (measurement conditions were dependent on studied bisphenol compound, details are discussed below). Properties of liquid samples were measured using quartz cuvette with a 1 cm path.

### 3.3. Chromatographic Method

HPLC system Varian 920-LC (Varian Inc., Mulgrave, Australia) was used for analyses. It was coupled with two detector types—a diode array detector and a ProStar 360 (Varian Inc., Mulgrave, Australia) fluorescence detector. DAD signals were registered from 200 to 400 nm (Δ = 1 nm). The fluorescence detector was set at 230 nm for excitation and 300 nm for emission with detector sensitivity set as medium.

The components were separated using an EC 250 mm × 4.6 mm NUCLEOSIL 100-5 C18 column (guard column EC 4/3 NUCLEOSIL 100-5 C18). An injection volume of 10 μL was used for each sample. The column temperature was set to 30 °C. Total duration of single analysis was 10 min.

**Figure 4:**
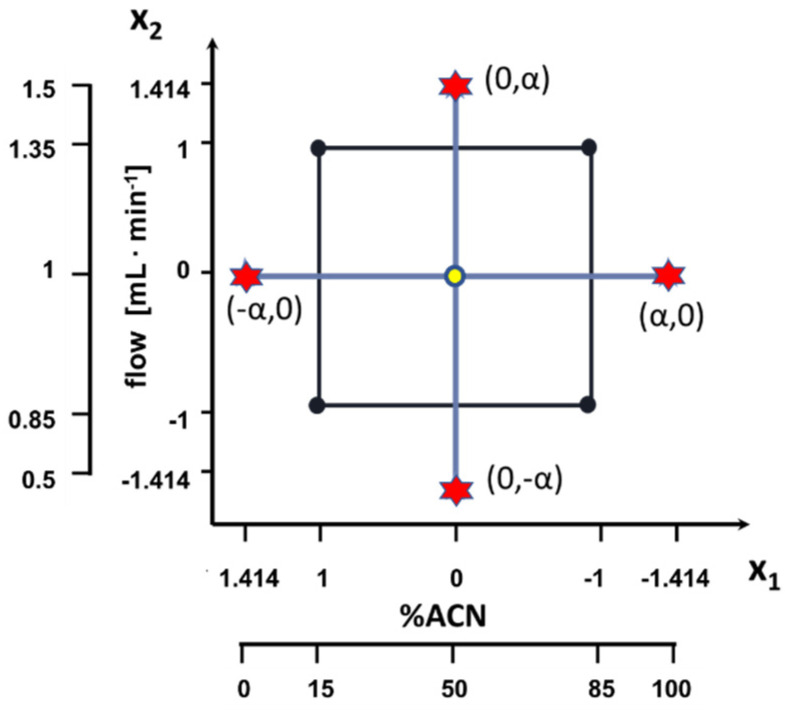
Design of the experiment.

Optimal mobile phase composition and flow rate for the chromatographic analysis were determined with a statistical approach. Conditions were optimized using the central composite design (CCD) with two factors (mobile phase composition and flow rate). For each factor five levels were considered. The ranges and coded levels of studied factors are presented in Table 4 and in Figure 4.

The response variable (y) was the quality of the registered chromatogram. The quality factor was the separation of BPs. It ranged from 1 (no separation) to 5 (excellent separation). It was evaluated on the basis of DAD chromatographic signals. The following quadratic model was considered

y = b_0_ + b_1_ x_1_ + b_2_ x_2_ + b_3_ x_1_^2^ + b_4_ x_2_^2^ + b_5_ x_1_x_2_ + ε (1)

Analysis of variance, ANOVA, was used to determine the significance of each regression coefficient of the equation and to check the adequacy of the final model.

### 3.4. Samples Preparation

The thermal paper samples were cut into 2 mm pieces. For each sample, 0.3 g were extracted to 9 mL of ethanol for 10 min in an ultrasound bath (35 kHz). Extracts were filtered with a syringe filter (nylon, 0.40 µm) and stored in a refrigerator for further analysis. The canned food samples (peas, corn and jack fruit) were drained. Collected effluents were centrifuged for 10 min (50,000 rpm), filtered with a syringe filter (nylon, 0.40 µm) and stored in a freezer (−30 °C) for further analysis.

## 4. Conclusions

Sixteen variants of analytical methods were developed and evaluated with the basic analytical parameters, to compare the HPLC methods and the molecular fluorescence spectroscopy methods of BPs detection. It is concluded the complexes of BPs with β-CD and m-β-CD increase the fluorescence properties of all analyzed BPs. For BPA and BPF detection, m-β-CD complexes result in the highest sensitivity and the lowest LODs values. For the BPS detection, β-CD and m-β-CD increase sensitivity similarly, thus, cheaper β-CD can be used in routine measurements. Qualitative detection of BPs in real samples confirmed similar outcomes of studied approaches. The HPLC coupled with DAD or fluorescence detector results in methods had better analytical performance. Additionally, the simultaneous detection of BPA and BPF using the fluorescence approach is impossible due to similar spectral properties. However, considering routine applications, evaluation of BSs concentration is less time and cost consuming when performed with the fluorescence measurements than with the chromatographic approach. Both approaches produce a similar amount of chemical waste. However, the chromatographic method results in a higher percentage of harmful organic chemical waste (due to 50% acetonitrile in the mobile phase).

## Figures and Tables

**Table 1:** The ANOVA results.

Source of Variation	Sum of Squares	Degrees of Freedom	Mean Squares	*F*-Value	*p*-Value
x_1_	3.643	1	3.640	6.356	0.065
x_2_	5.625	1	5.620	9.814	0.035
x_1_^2^	45.0	1	45.0	78.580	0.001
x_2_^2^	5.0	1	5.0	8.731	0.042
x_1_ x_2_	0.001	1	0.001	0.002	0.969
Model	152.0	4	38.0	66.356	0.001
Residuals	8.586	15	0.573		

**Table 2:** Analytical parameters of developed methods.

System	Analytical Technique	Spectral Information *	Slope	Intercept	r	LOD ** [μg∙mL^−1^]	RSD ***	Linear Range [μg∙mL^−1^]
BPA	MSF	Ex = 235 nm Em = 300 nm	26.35	22.64	0.998	0.14	3.8%	0–2.4
BPA + β-CD	MSF	Ex = 235 nm Em = 300 nm	132.41	45.68	0.999	0.13	3.1%	0–2.8
BPA + m-β-CD	MSF	Ex = 235 nm Em = 300 nm	308.84	124.72	0.998	0.38	2.3%	0–2.8
BPA	HPLC-DAD	275 nm	6.3 × 10^3^	10.16	0.999	0.04	-	0–1125
BPA	HPLC-FLD	Ex = 230 nm Em = 300 nm	1.56 × 10^5^	1.88∙× 10^4^	0.999	0.07	-	0–3.5
BPF	MSF	Ex = 235 nm Em = 304 nm	55.91	22.93	0.999	0.60	4.8%	0–2.8
BPF + β-CD	MSF	Ex = 235 nm Em = 304 nm	106.28	61.26	0.991	0.35	2.8%	0–2.8
BPF + m-β-CD	MSF	Ex = 235 nm Em = 304 nm	259.3	70.76	0.999	0.11	2.6%	0–2.8
BPF	HPLC-DAD	275 nm	6.26 × 10^3^	18.98	0.999	0.04	-	0–1125
BPF	HPLC-FLD	Ex = 230 nm Em = 300 nm	1.49 × 10^5^	1.45 × 10^4^	0.999	0.10	-	0–3.5
BPS	MSF	Ex = 335 nm Em = 400 nm	0.61	19.31	0.986	1.82	4.3%	0–15
BPS + β-CD	MSF	Ex = 335 nm Em = 400 nm	14.76	29.17	0.997	0.84	4.3%	0–15
BPS + m-β-CD	MSF	Ex = 335 nm Em = 400 nm	14.77	32.33	0.997	0.83	3.0%	0–15
BPS	MSF	Ex = 295 nm Em = 471 nm	0.31	18.52	0.986	8.30	5.7%	0–180
BPS + β-CD	MSF	Ex = 295 nm Em = 471 nm	0.36	24.81	0.997	15.44	4.9%	0–300
BPS + m-β-CD	MSF	Ex = 295 nm Em = 471 nm	0.45	17.96	0.997	7.35	2.8%	0–300
BPS	HPLC-DAD	225 nm	1.38 × 10^4^	167.95	0.999	0.04	-	0–1125

* Ex stands for excitation wavelength, Em stands for emission wavelength. ** The LODs were computed according to the formula: LOD = (3.3 × s_b_) × b^−1^, where, b is an intercept of a linear regression equation and s_b_ is its standard deviation. *** The RSDs were computed according to the formula: RSD = s_z_ × 100% × $\;\bar{z}\;$^−1^, where, s_z_ (standard deviation) and $\;\bar{z}$ (mean) were computed for a batch of six samples (1.2 μg∙mL^−1^).

**Table 3:** Concentration of BPs in studied samples obtained from different analytical methods.

		HPLC-DAD	HPLC-FLD	MFS
Sample	BP	Concentration * [μg∙g^−1^] or [μg∙mL^−1^]		
TP no. 1	BPS	2. 8 ± 0.05	-	3.84 ± 0.30
TP no. 1	BPF	not detected	6.49 ± 1.00	6.28 ± 0.35
TP no. 2	BPS	2.28 ± 0.05	-	3.13 ± 0.15
TP no. 3	BPF	10.04 ± 0.03	10.78 ± 0.03	12.05 ± 0.38
Peas	BPA	12.35 ± 0.07	13.05 ± 0.06	13.61 ± 0.12
Corn	BPA	18.21 ± 0.02	17.91 ± 0.02	20.61 ± 0.35
Jack fruit	BPA	28.26 ± 0.04	26.41 ± 0.05	27.64 ± 0.25

* For TP samples values expressed as μg∙g^−1^, for canned food samples values expressed as μg∙mL^−1^. The ± values are standard deviations obtained for three sample replicates.

**Table 4:** Design of the experiment.

Oryginal		Coded					
x_1_ (%ACN)	x_2_ (Flow [mL∙min^−1^])	x_0_	x_1_	x_2_	x_1_^2^	x_2_^2^	x_1_x_2_
85	0.85	1	1	−1	1	1	−1
85	0.85	1	1	−1	1	1	−1
15	0.85	1	−1	−1	1	1	1
15	0.85	1	−1	−1	1	1	1
85	1.35	1	1	1	1	1	1
85	1.35	1	1	1	1	1	1
15	1.35	1	−1	1	1	1	−1
15	1.35	1	−1	1	1	1	−1
100	1	1	1.414	0	2	0	0
100	1	1	1.414	0	2	0	0
50	0.5	1	0	−1.414	0	2	0
50	0.5	1	0	−1.414	0	2	0
50	1.5	1	0	1.414	0	2	0
50	1.5	1	0	1.414	0	2	0
0	1	1	−1.414	0	2	0	0
0	1	1	−1.414	0	2	0	0
50	1	1	0	0	0	0	0
50	1	1	0	0	0	0	0
50	1	1	0	0	0	0	0
50	1	1	0	0	0	0	0

## Supplementary Materials

The following are available online at www.mdpi.com/article/10.3390/ijms221910569/s1, Figure S1: Absorption spectra of selected bisphenols and their equimolar complexes with β-CD and m-β-CD in phosphate buffer (pH = 7.00) for (a) BPA, (b) BPF, and (c) BPS. Figure S2. Emission spectra of selected bisphenols and their equimolar complexes with m-β-CD under pH 2–10 for (a) BPA, (b) BPF, and (c) BPS. Figure S3: Job’s plots of equimolar solutions of BPs and CDs compounds for: (a) BPA + β-CD (Excitation = 235 nm, Emission = 300 nm), (b) BPA + m-β-CD (Excitation = 235 nm, Emission = 300 nm), (c) BPF + β-CD (Excitation = 235 nm, Emission = 304 nm), (d) BPF + m-β-CD (Excitation = 235 nm, Emission = 304 nm), (e) BPS + β-CD (Excitation = 295 nm, Emission = 471 nm), (f) BPS + m-β-CD (Excitation = 295 nm, Emission = 471 nm). Figure S4: Calibration curves for: (a) BPA systems, excitation 235 nm, emission 300 nm; (b) BPF systems, excitation 235 nm, emission 304 nm, (c) BPS systems, excitation 355 nm, emission 400 nm; (d) BPS systems excitation 295 nm emission 471 nm.
