# Supplementary material for: Comparison of Quantitative Detection Methods Based on Molecular Fluorescence Spectroscopy and Chromatographic Techniques Used for the Determination of Bisphenol Compounds
Source: Int J Mol Sci. 2021 Sep 29;22(19):10569. doi: 10.3390/ijms221910569 (PMC8508877; doi:10.3390/ijms221910569)
Supplement: Supplementary file 1 [file ijms-22-10569-s001.zip › ijms-1348612-supplementary.pdf]

International Journal of
Molecular Sciences

MDPI

Supplementary materials

a)

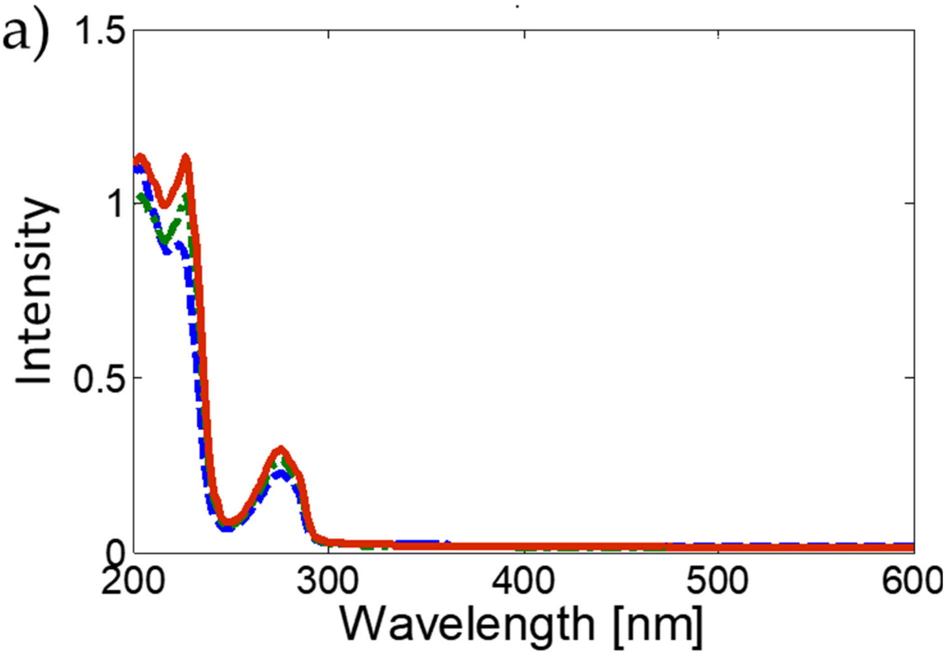

b)

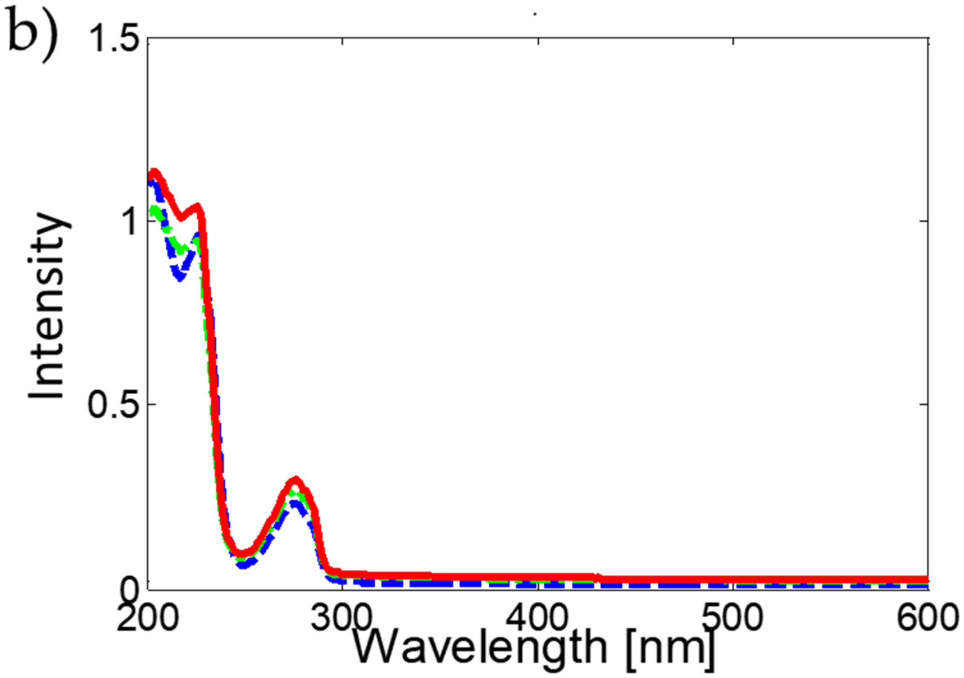

c)

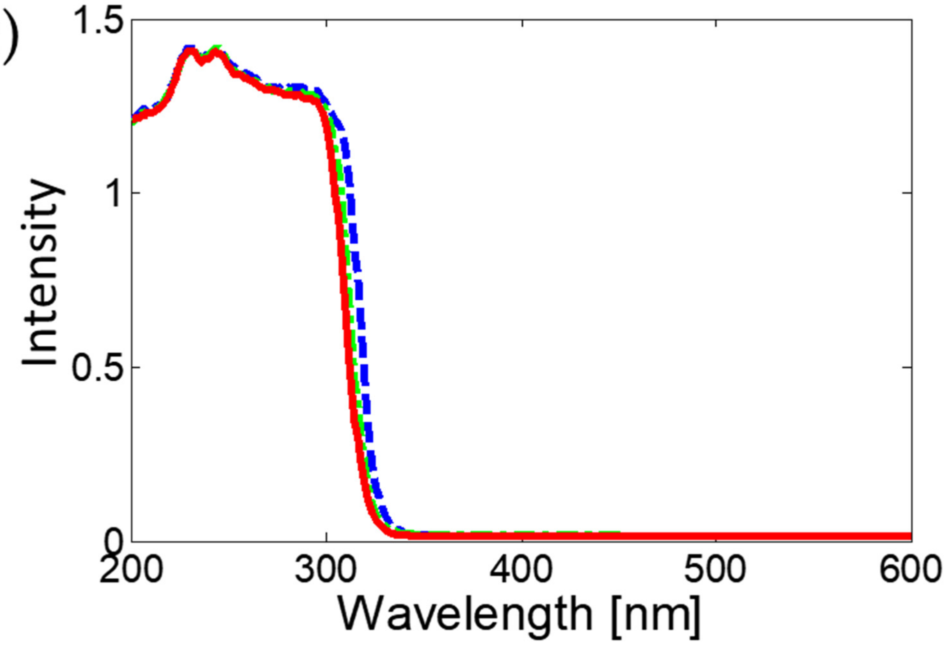

Figure S1. Absorption spectra of selected bisphenols and their equimolar complexes with β-CD and m-β-CD in phosphate buffer (pH=7.00) for (**a**) BPA, (**b**) BPF, and (**c**) BPS.

a)

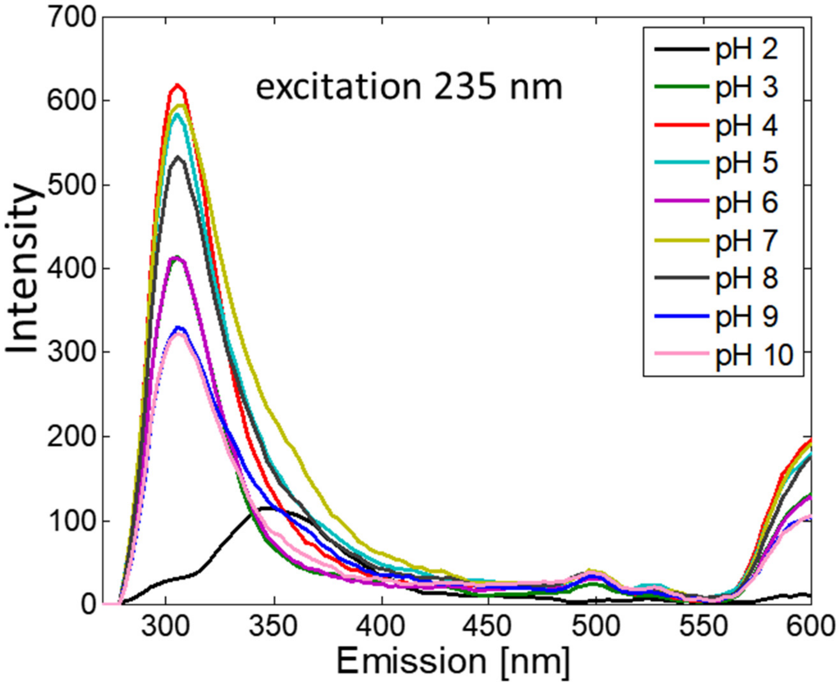

b)

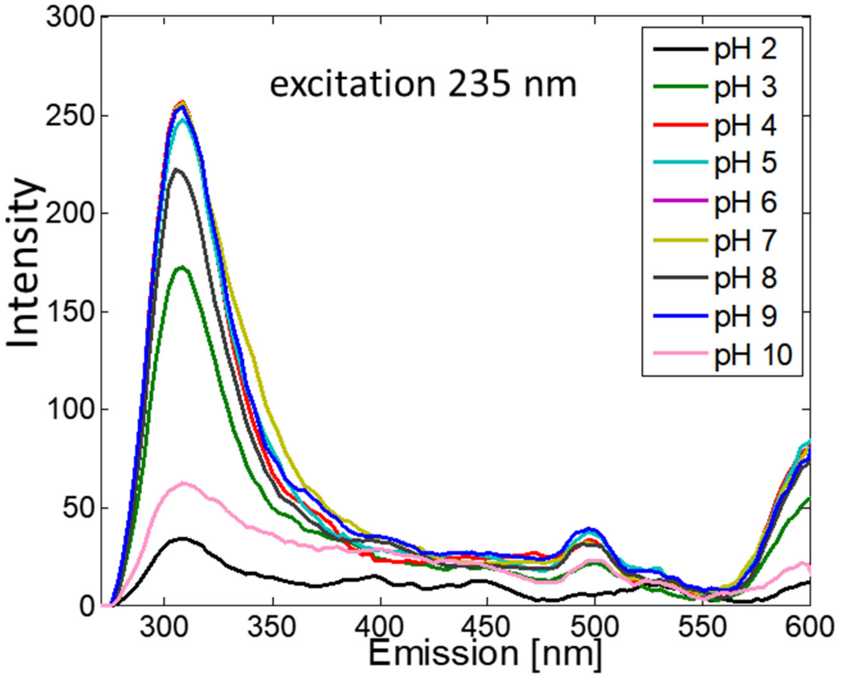

c)

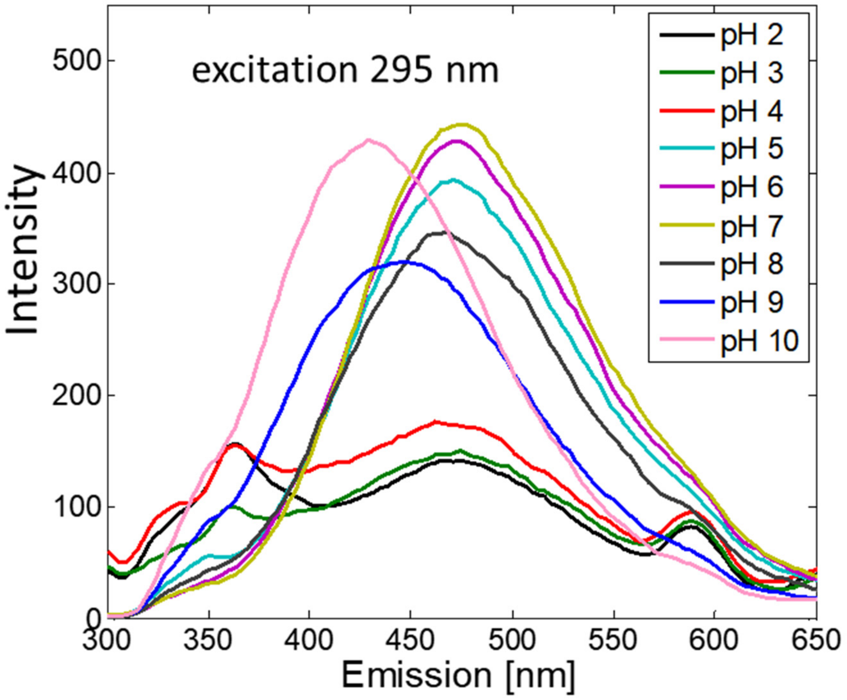

Figure S2. Emission spectra of selected bisphenols and their equimolar complexes with m-β-CD under pH 2–10 for (**a**) BPA, (**b**) BPF, and (**c**) BPS.

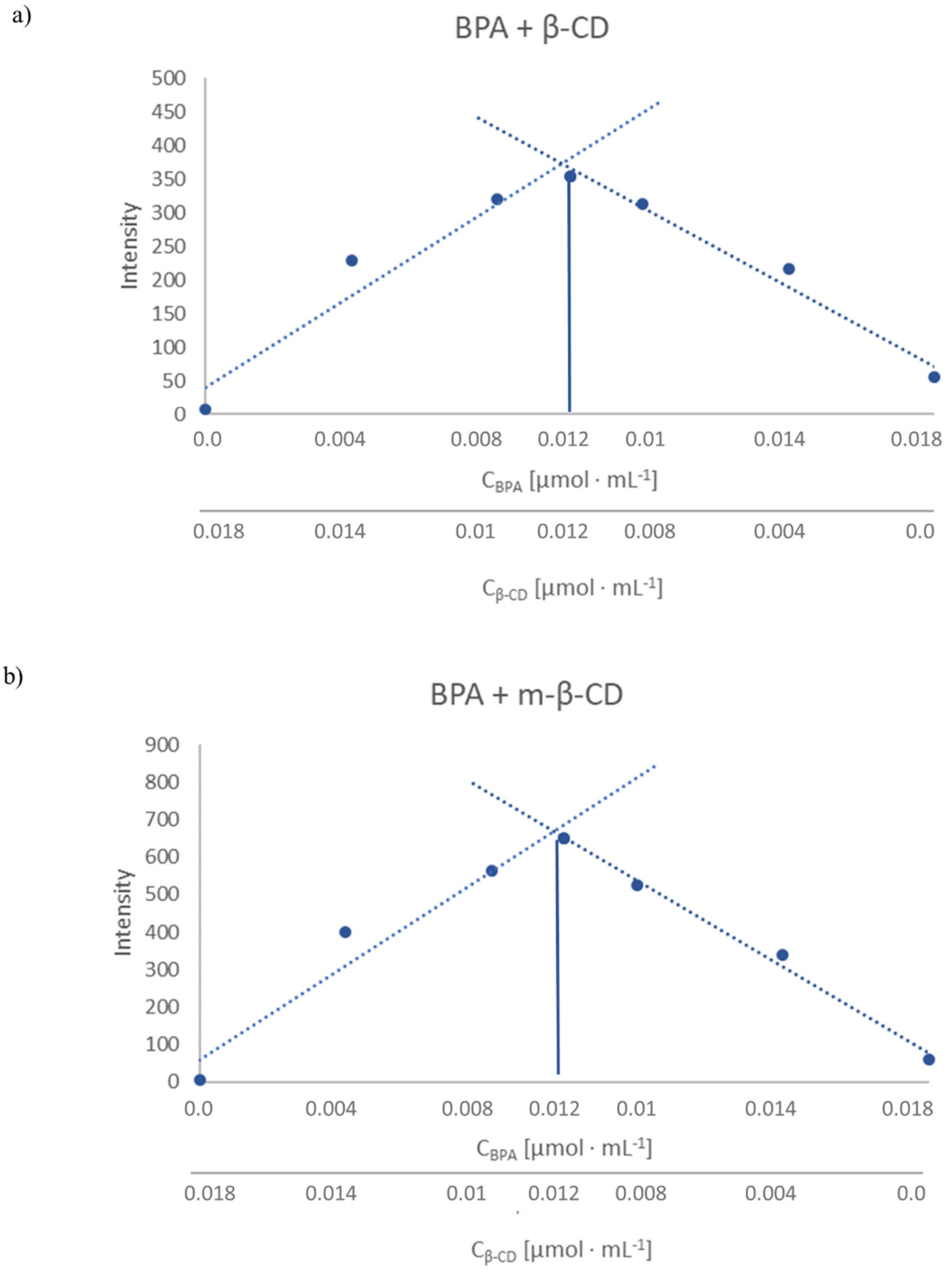
a)
BPA + β-CD
Intensity
500
450
400
350
300
250
200
150
100
50
0
0.0 0.004 0.008 0.012 0.01 0.014 0.018
C BPA [µmol · mL-1]
0.018 0.014 0.01 0.012 0.008 0.004 0.0
C β-CD [µmol · mL-1]
b)
BPA + m-β-CD
Intensity
900
800
700
600
500
400
300
200
100
0
0.0 0.004 0.008 0.012 0.01 0.014 0.018
C BPA [µmol · mL-1]
0.018 0.014 0.01 0.012 0.008 0.004 0.0
C β-CD [µmol · mL-1]

c)

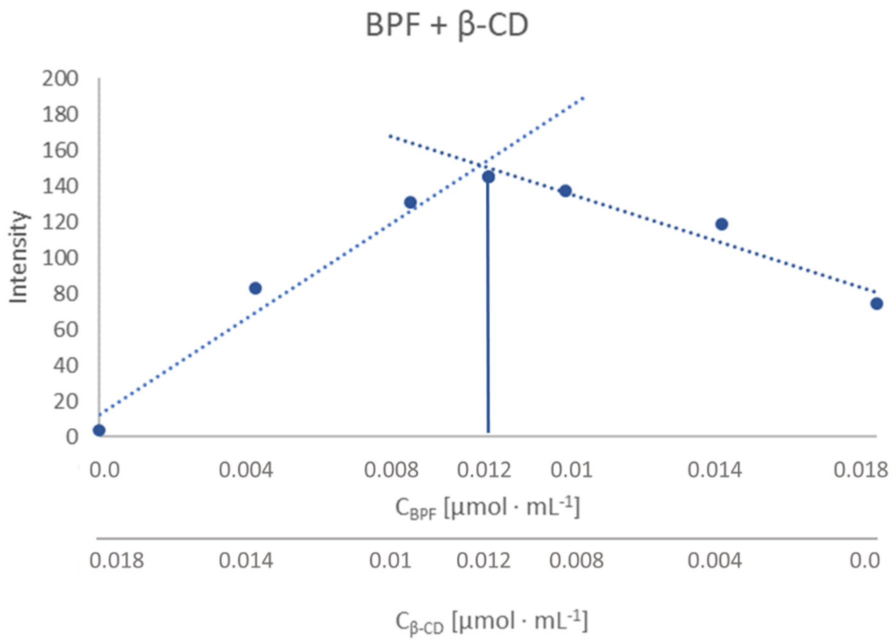
BPF + β-CD
Intensity
200
180
160
140
120
100
80
60
40
20
0
0.0
0.004
0.008
0.012
0.01
0.014
0.018
CBPF [µmol · mL-1]
0.018
0.014
0.01
0.012
0.008
0.004
0.0
Cβ-CD [µmol · mL-1]

d)

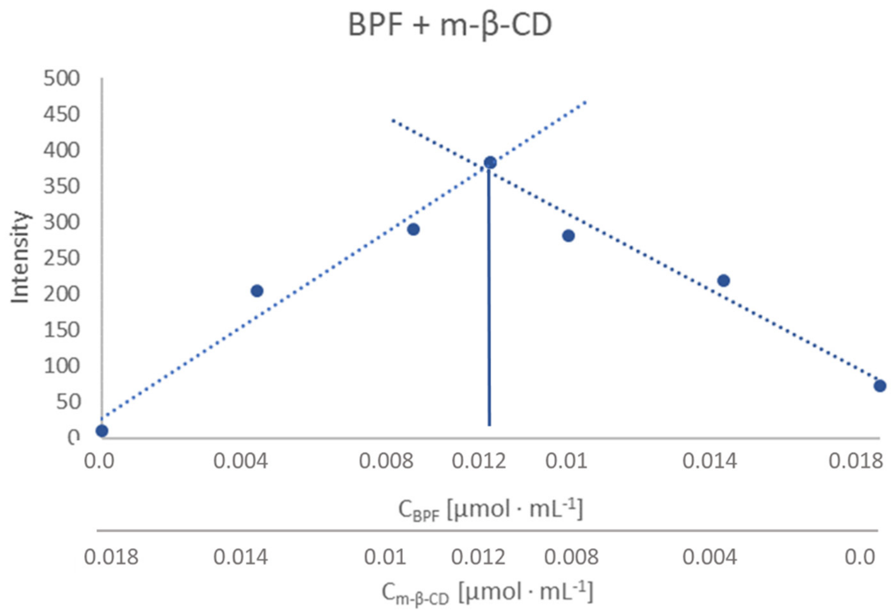
BPF + m-β-CD
Intensity
500
450
400
350
300
250
200
150
100
50
0
0.0
0.004
0.008
0.012
0.01
0.014
0.018
CBPF [µmol · mL-1]
0.018
0.014
0.01
0.012
0.008
0.004
0.0
Cm-β-CD [µmol · mL-1]

e)

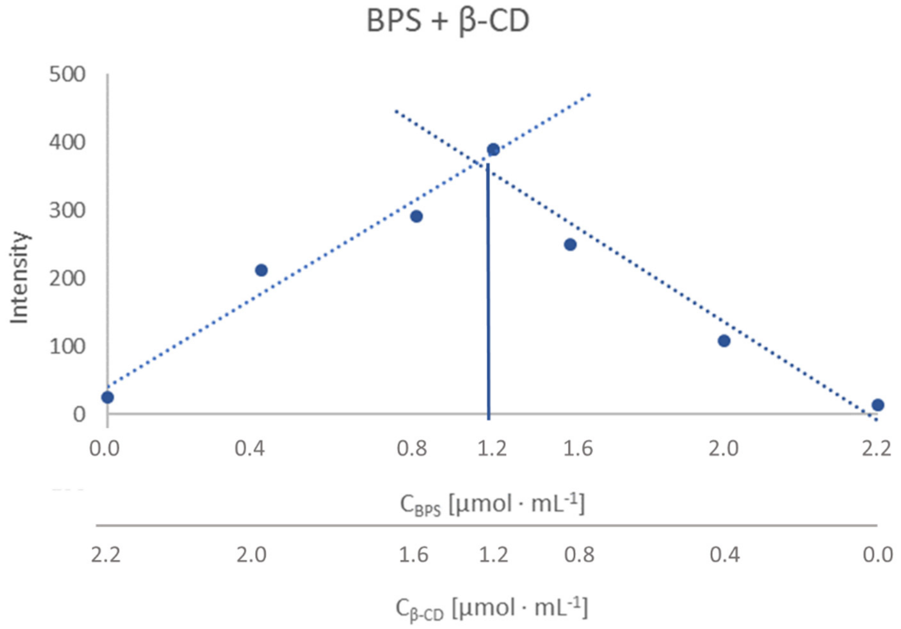

f)

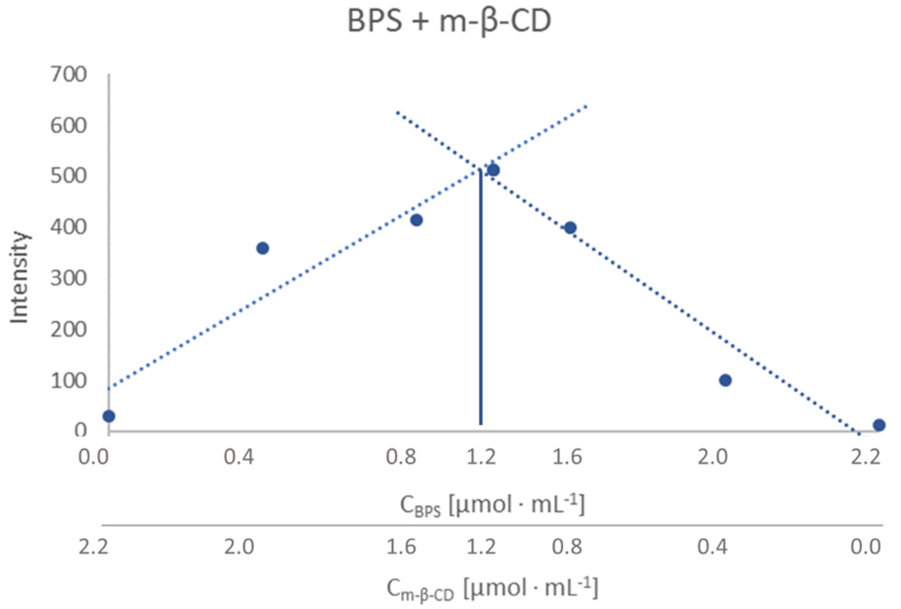

Figure S3. Job's plots of equimolar solutions of BPs and CDs compounds for: (**a**) BPA + β-CD (Ex-citation = 235 nm, Emission = 300 nm), (**b**) BPA + m-β-CD (Excitation = 235 nm, Emission = 300 nm), (**c**) BPF + β-CD (Excitation = 235 nm, Emission = 304 nm), (**d**) BPF + m-β-CD (Excitation = 235 nm, Emission = 304 nm), (**e**) BPS + β-CD (Excitation = 295 nm, Emission = 471 nm), (**f**) BPS + m-β-CD (Excitation = 295 nm, Emission = 471 nm).

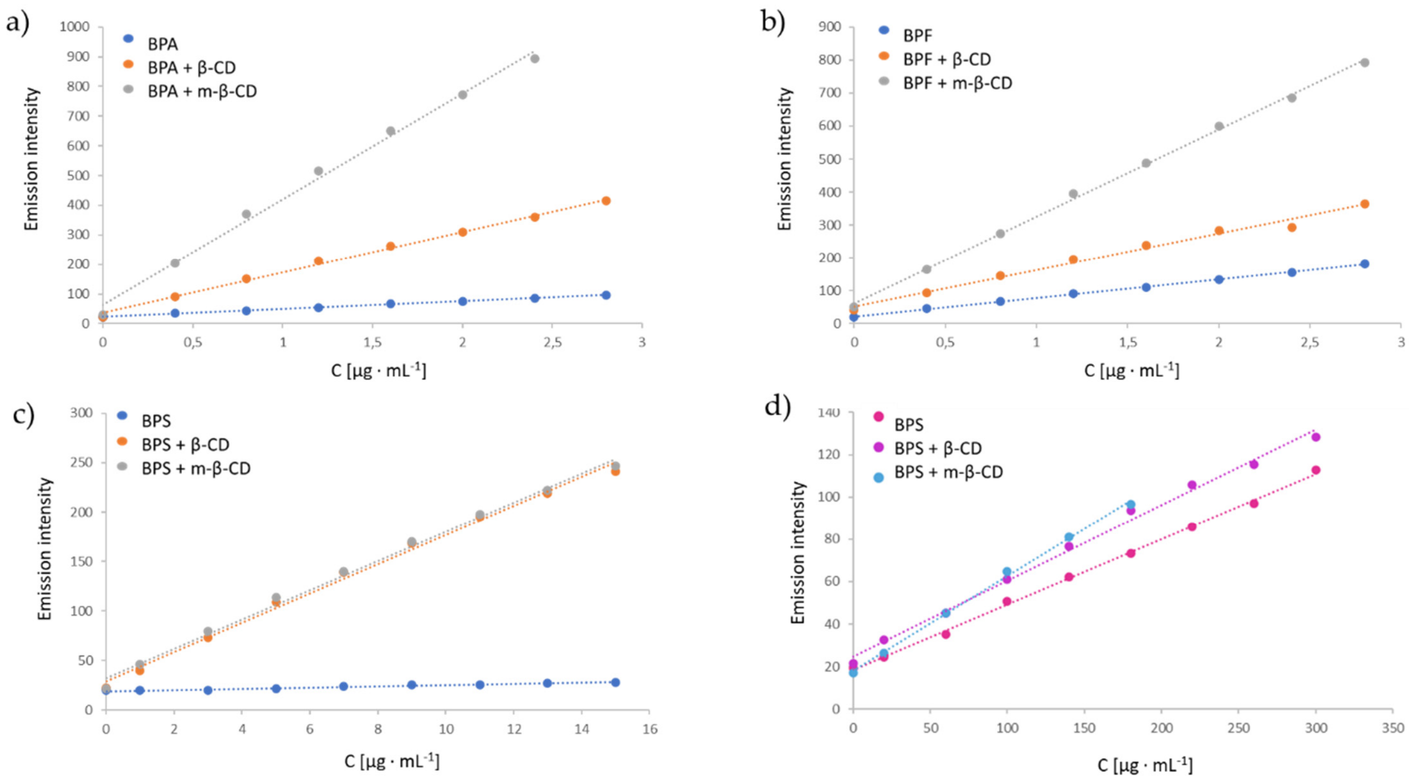

Figure S4. Calibration curves for: (**a**) BPA systems, excitation 235 nm, emission 300 nm; (**b**) BPF systems, excitation 235 nm, emission 304 nm, (**c**) BPS systems, excitation 355 nm, emission 400 nm; (**d**) BPS systems excitation 295 nm emission 471 nm.
